# Multi‐ and Gray‐Scale Thermal Lithography of Silk Fibroin as Water‐Developable Resist for Micro and Nanofabrication

**DOI:** 10.1002/advs.202303518

**Published:** 2024-01-17

**Authors:** Mohammadreza Rostami, Aleksandra Marković, Ya Wang, Joffrey Pernollet, Xiaosheng Zhang, Xia Liu, Juergen Brugger

**Affiliations:** ^1^ Microsystems Laboratory Ecole Polytechnique Fédérale de Lausanne (EPFL) Lausanne 1015 Switzerland; ^2^ Center for Micro and Nanotechnology (CMi) Ecole Polytechnique Fédérale de Lausanne (EPFL) Lausanne 1015 Switzerland; ^3^ School of Electronic Science and Engineering University of Electronic Science and Technology of China (UESTC) Chengdu 611731 China; ^4^ School of Integrated Circuits and Electronics MIIT Key Laboratory for Low‐Dimensional Quantum Structure and Devices Beijing Institute of Technology Beijing 100081 China; ^5^ Current affiliation: Food Science and Technology Program, Department of Life Sciences BNU‐HKBU United International College Zhuhai 519087 China

**Keywords:** direct write laser (DWL), dry etching, silk fibroin (SF), solubility change, thermal scanning probe lithography (t‐SPL), water development

## Abstract

Silk fibroin (SF) is a natural material with polymorphic structures that determine its water solubility and biodegradability, which can be altered by exposing it to heat. Here, a hybrid thermal lithography method combining scalable microscale laser‐based patterning with nanoscale patterning based on thermal scanning probe lithography is developed. The latter enables in addition grayscale patterns to be made. The resolution limit of the writing in silk fibroin is studied by using a nanoscale heat source from a scanned nanoprobe. The heat thereby induces local water solubility change in the film, which can subsequently be developed in deionized water. Nanopatterns and grayscale patterns down to 50 nm lateral resolution are successfully written in the silk fibroin that behaves like a positive tone resist. The resulting patterned silk fibroin is then applied as a mask for dry etching of SiO_2_ to form a hard mask for further nano‐processing. A very high selectivity of 42:1 between SiO_2_ and silk fibroin is obtained allowing for high‐aspect ratio structure to be fabricated. The fabricated nanostructures have very low line edge roughness of 5 ± 2 nm. The results demonstrate the potential of silk fibroin as a water‐soluble resist for hybrid thermal lithography and precise micro/nanofabrication.

## Graphical abstract

This study presents a hybrid thermal lithography method combining scalable microscale patterning based on direct‐write laser with nanoscale/ grayscale patterning based on thermal scanning probe lithography. This study demonstrates that the bio‐polymer silk fibroin shows excellent characteristics as positive tone resist and can be developed in DI water aiming for high‐resolution green‐nanomanufacturing.

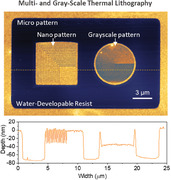

## 1 Introduction

SF has attracted great interest as a biocompatible resist^[^ ^1^, ^2^ ^]^ in the past years due to its remarkable mechanical strength, high optical transparency (>95% in the visible range) and tunable biodegradability.^[^ ^3^, ^4^, ^5^ ^]^ Silk is a natural material produced by certain spiders such as *Nephilia clavipes* or worms such as the domestic silkworm *Bombyx mori*. Silk from *Bombyx mori* is composed of fibroin surrounded by a hydrophilic protein sericin, which, for technical applications, is typically removed during a degumming process.^[^ ^6^ ^]^ SF can stabilize via inter‐chain hydrogen bond interactions to form α‐helix structures and β‐sheet secondary structures. Its polymorphic property lets SF appear in at least two dissimilar structures, i) the so‐called silk I (type II β‐turn) and ii) the so‐called silk II (antiparallel β‐sheet).^[^ ^7^, ^8^ ^]^ SF prepared from aqueous solution and subsequently dried in air on a substrate is composed mainly of a random coil/α‐helix conformation, which is the amorphous phase and is water‐soluble.^[^ ^9^ ^]^ A phase change from the amorphous phase to crystalline phase that becomes water‐insoluble can be induced by treatment with organic solvents such as ethanol or methanol to promote the formation of beta‐sheet crystallites, a process also termed as annealing. The crystallites act as cross‐linkers between fibroin strands and prevent the material from being dissolved in water.^[^ ^10^ ^]^

SF can be effectively functionalized through appropriate doping, allowing for preservation of activities of the entrapped dopants, which has great potential in bio‐electronics and biosensing applications. SF has attracted considerable attention in various research areas, including optical diffraction gratings,^[^ ^11^, ^12^ ^]^ organic transistors,^[^ ^13^, ^14^ ^]^ volatile memristors for neuromorphic computing,^[^ ^15^ ^]^ biosensors,^[^ ^14^, ^16^ ^]^ and photonic devices.^[^ ^17^ ^]^ Structuring of SF is required to obtain desired shapes and functionalities of these devices. Micrometer sized SF patterns have been fabricated by soft‐lithography‐based molding,^[^ ^2^ ^]^ rapid thermal nanoimprinting,^[^ ^17^, ^18^ ^]^ laser ablation,^[^ ^19^, ^20^, ^21^, ^22^, ^23^, ^24^ ^]^ templating,^[^ ^25^ ^]^ UV photolithography,^[^ ^26^, ^27^, ^28^ ^]^ and multiphoton lithography.^[^ ^29^, ^30^ ^]^ Nanoscale patterning of SF by electron beam lithography and photolithography is based either on the decomposition of the protein, such as by phototendering,^[^ ^31^ ^]^ electron irradiation,^[^ ^29^, ^32^, ^33^ ^]^ the formation of chemical crosslinks through the addition of a photoinitiator^[^ ^34^ ^]^ or functionalization of the protein.^[^ ^16^ ^]^ In addition, β‐sheet crystallites can be molten by fast scanning chip calorimetry using a very high heating rate up to 2000 K s^−1^ with little thermal degradation of the SF.^[^ ^35^, ^36^, ^37^ ^]^ However, this melting transition does not materialize kinetically at low heating rates, since thermal decomposition and mass loss occur at a temperature lower than the melting temperature of β‐pleated sheet crystallites. Therefore, the application of this SF resist in hybrid thermal lithography for microscale, nanoscale and grayscale fabrications is unexplored because up to date no known patterning technique is capable of exposing SF at the sub‐micrometer scale to the required heat with the necessary very high heating and cooling rates.

Advanced direct‐write laser (DWL) lithography and the recently developed thermal scanning probe lithography (t‐SPL) exhibit unique capabilities to locally expose a thin film to high temperature with ultra‐high heating (and cooling) rates (≈10^8^ K s^−1^). Indeed, combining DWL with t‐SPL in a single lithography tool has enabled novel mix and match lithography. One of the distinct advantages is its multi‐scale capability, where the laser is used to directly expose (and ablate) the polymer limited by the diffraction of the optical system and wavelength, and where the adjacent t‐SPL probe exposes the same polymer to heat with ≈10 nm resolution.^[^ ^38^, ^39^ ^]^ In t‐SPL patterns are formed by consecutive indentation with the heated probe for a period of 1–20 µs per indent which can be subsequently imaged by the same, cooled‐down probe in a closed loop. In this article, we demonstrate that the SF shows excellent characteristics as positive tone resists for thermal micro and nano lithography by combining both DWL and t‐SPL for multi‐scale and grayscale patterning. In addition, the patterned SF film shows outstanding performance when applied as a hard mask for dry etching of sub‐50 nm structures with a very high selectivity of 42:1 between SiO_2_ and SF.

## 2 Results and Discussion

**Figure 1:**
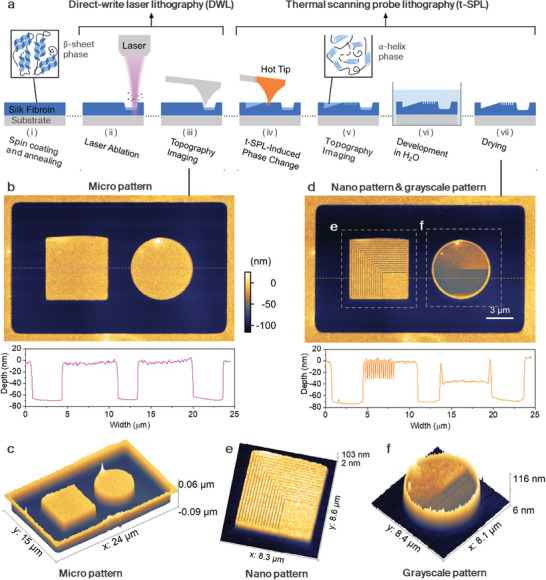
Hybrid thermal lithography with silk fibroin as a positive resist. a) Process flow of the lithography enabling large‐area micropatterns written by DWL and nanopatterns and grayscale patterns written by t‐SPL. After patterning, the thermally modified resist (as drawn in light blue in step ii–v) is dissolved by development in DI water. b) Topography image of micropatterns created by DWL (before water development) and the corresponding profile measured by the t‐SPL probe. The thickness of the resist is 100 nm and the pattern depth is ≈70 nm. c) Corresponding 3D topography image of panel b. d) AFM image of the nanopattern and the grayscale pattern by t‐SPL overlaying with the pattern in panel b which shows resolution down to 150 nm and alignment accuracy in the order of 100 nm. The profile below corresponds to the cross‐section of the nano pattern and grayscale pattern. The depth of the nanopattern is 30 nm. The depth of the grayscale pattern is in the range of 30–50 nm. e,f) 3D view of the topography images of the selected areas in panel d.

Preparing the SF film follows a procedure reported elsewhere.^[^ ^40^ ^]^ Patterning of the annealed SF films is performed using a commercial laser‐ and probe‐based micro‐ and nanopatterning system (NanoFrazor Explore, Heidelberg Instruments Nano) equipped with both a t‐SPL and an integrated DWL having a 405 nm wavelength laser source as shown in **Figure** **1**. Figure 1a shows the working principle of the micro‐/nano‐patterning on the SF resist using the hybrid thermal lithography with subsequent development in DI water. First, the laser source is used to write micropatterns with an area of 25 × 15 µm by either thermal ablation with high laser power or thermal‐induced effect with low laser power. Figure 1b shows the topography image taken with the t‐SPL probe in the reading mode of the micropattern made by means of laser ablation from DWL (laser power of 219 mW, exposure time of 50 µs per indent). The initial SF film is 100 nm thick, and the depth of the direct laser written pattern is ≈70 nm as shown in the topography image. This indicates that the bottom SF layer of 30 nm thick is remained by the laser ablation. Please note that at this point of time, we have not yet performed a DI water development step (Figure 1b bottom). Next, the heated probe is used for patterning the SF film at nanoscale due to the local thermal‐induced phase change. Subsequently, we perform a DI water development step at room temperature for 30 s and blow dry the sample with a N_2_ gun. Figure 1d shows an AFM microscope image of three patterning results that are in proximity next to each other, after DI water development: i) the laser written micropattern, ii) the t‐SPL written nanopattern and iii) the grayscale pattern. These results clearly show the unique potential in combination of DWL and t‐SPL for excellent overlay between micropatterns and nanopatterns, written by different techniques. Additionally, the cross‐sectional SEM images of the SF structures patterned by DWL and t‐SPL show sharp edges with low surface roughness in Figure S1 (Supporting Information). The high accuracy comes from the intrinsic capability of integrated probe alignment to a pre‐existing (or as previously created in our case) surface topography. Therefore, the SF resist can be used for hybrid thermal lithography with a very wide range of pattern dimensions and high alignment accuracy. The DWL is typically used for large size, micrometer patterns, whereas the t‐SPL is used for high‐resolution nanometer patterns near the larger structures, thus also saving overall writing time. The depth of the t‐SPL written nanopattern after DI water development was measured to be 30 nm and the depth of the grayscale pattern is in the range of 30–50 nm as shown in Figure 1e,f. The grayscale pattern is achieved by adjusting the indentation depth and/or writing temperature. A wide range of grayscale patterns can be achieved having a linear gradient from 30 to 200 nm over an 18 × 5 µm region as shown in Figure S2 (Supporting Information).

**Figure 2:**
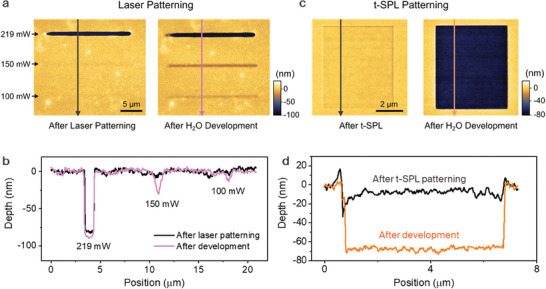
Analysis of the pattern mechanisms of the SF resist by the hybrid thermal lithography. a) Topography images of line patterns written by DWL with different laser powers before and after subsequent development in DI water. The line patterns written with a laser power of 100 and 150 mW become only clearly visible after the DI water development. b) Corresponding profiles show that the pattern depths vary with the laser power and the corresponding depth increases after the development. c) Topography images of patterns created by t‐SPL where a 6 × 6 µm square is exposed to the hot tip with a heater temperature of 800 °C, heat pulses of 20 µs and after the development in DI water for 30 s. d) Corresponding profiles show that the t‐SPL thermal effect cannot ablate the SF film but local phase change using the hot tip renders the exposed resist water soluble.

The dependence of DWL patterning under varying laser power is investigated and the results are shown in **Figure** **2**. As shown in Figure 2a, on the one hand, after laser exposure with a high laser power of 219 mW and a pulse duration of 50 µs, a ≈75 nm thick layer of the resist is directly ablated from the substrate (Figure 2b). On the other hand, by using a low laser power of less than 150 mW the SF is barely exposed to heat and consequently undergoes a phase change, but the energy is not sufficient to ablate the film and no significant topography (<10 nm) change can be measured. The threshold laser power for directly patterning the resist decreases with longer exposure time as shown in detail in Section S3 (Supporting Information). We also notice that after water development the depth of the written pattern increases by ≈5 nanometers (Figure 2b). This is ascribed to the fact that the non‐ablated remaining silk has been exposed to laser energy and is thereby converted from β to α phase and consequently becomes water soluble in the subsequent DI water development step. Therefore, the patterning of SF using DWL depends on two mechanisms: i) direct laser ablation and ii) solubility change of remaining SF layer.

Next, we systematically study the patterning of the SF film by t‐SPL. To this end, we apply a heater temperature *T*_h_ of 800 °C (The actual tip temperature is ≈1/3 of the heater temperature).^[^ ^39^, ^41^, ^42^ ^]^ We apply heat pulses of 20 µs which result in shallow SF surface indentations of < 10 nm as seen in the left image of Figure 2c which are caused by the tip indentation and thermal‐induced effect on the SF (i.e., residual water evaporation).^[^ ^43^ ^]^ After developing the patterned SF film in DI water for 30 s, the thermally exposed part is dissolved as shown in the topography contrast where a depth of ≈70 nm is now measured in the profile (right image in Figure 2c). The corresponding profiles plotted in Figure 2d show a significant difference in the pattern depths between the t‐SPL patterning and the development. Furthermore, the pattern depth increases with higher *T*_h_ up to 120 nm, as discussed in detail in the Section S4 (Supporting Information).

**Figure 3:**
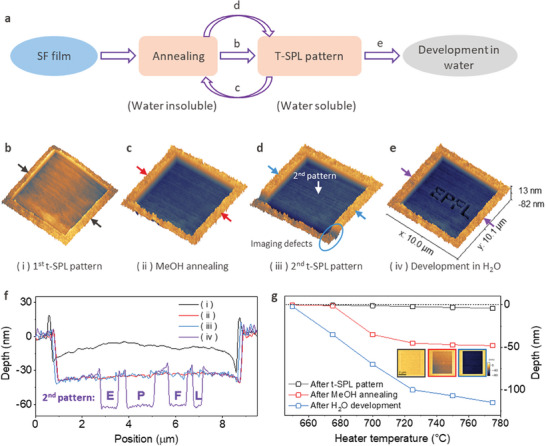
Repeatable reversibility of phase change in the silk fibroin. a) Illustration of repeatability of reversible phase change of silk fibroin. b–e) Patterning a square on the annealed silk fibroin resist, annealing the patterned area in methanol, patterning the EPFL logo on the annealed square, and development in water, respectively. f) Surface profiles of 1st patterned SF, annealed patterned SF, 2nd patterned SF, and developed SF as indicated by the line in panels b, c, d, and e, respectively. g) The pattern depth is plotted as a function of the heater temperature after t‐SPL, methanol immersion for 10 min and after water development for 30 s. The inset is the images of surface topography of the silk fibroin film after t‐SPL pattern, after methanol annealing and after water development. The color contrast of the pattern square shows the variation of the depth.

Another characteristic of silk fibroin is that it can be reversibly switched back and forth between the two phases by applying annealing ( = crystallization) and heating ( = amorphization) steps. We are exploring this property by the following experiment, shown in **Figure** **3**. First, we use t‐SPL (*T*_h_ = 700 °C) to write a square area of 8 × 8 µm in a spin‐coated SF film that we previously annealed in methanol. Second, an annealing step is performed in pure methanol for 10 min to form β‐sheet‐phase SF in the thermally written area, thereby decreasing the thickness of the patterned square by 25 nm (Figure 3c,f). This is due to the dissociation of a thin surface layer of the thermally treated SF during immersion in methanol (Figure 3e,f). Third, the t‐SPL is used again to write a new nanopattern (EPFL logo) (Figure 3d) inside the previously annealed/written/annealed square pattern which results in a marginal change of topography (Figure 3f). It is noted that the depths of the square pattern, excluding the areas heated by the 2nd t‐SPL in the step (ii) and the step (iv) are similar as shown in Figure 3f. It means that the 1st patterned square is not dissolved in water in the step (iv), indicating that certain phase change from water‐soluble and water‐insoluble phases occurs in MeOH annealing, as illustrated in details in Supplementary Figure S5 (Supporting Information). And finally, the structure is developed in DI water which reveals the deeper nanopattern inside the shallow micropattern. In other words, the patterned area indeed becomes water insoluble again after the second annealing treatment. This successful re‐patterning shows that the SF resist can be repeatably changed between water‐soluble and water‐insoluble phases, if the phase transition is sufficiently gentle (not too high temperature and not too long exposure time that would damage the polymer), and thus it can be patterned again after reannealing.

We repeated this experiment for various heater temperatures between 650 and 775 °C as shown in Figure 3g to investigate the temperature dependence of the amorphization. Up to 650 °C, neither a visible pattern is created, nor material dissolves in methanol or water. At 675 °C, the pattern becomes visible after t‐SPL but only a very thin layer of SF is dissolved during methanol annealing. After immersion in DI water the heat exposed material is dissolved resulting in a 45 nm deep pattern. While increasing the probe temperature, the pattern depth after development increases up to 110 nm, which is attributed to the increased temperature around the tip. Above 725 °C, the depth after methanol immersion does not show further change as function of the probe temperature.

**Figure 4:**
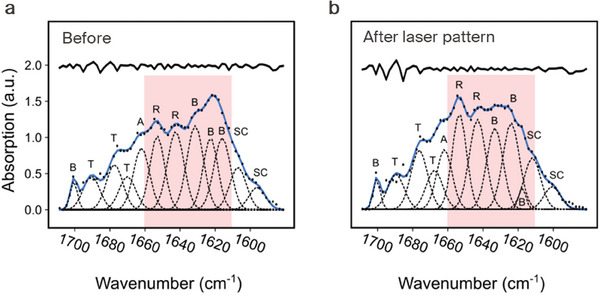
FTIR analysis of composition of the silk fibroin resist before and after laser patterning. a) FTIR analysis of the annealed silk fibroin before laser exposure. b) After laser exposure, the peaks ≈1620 cm^−1^ are reduced on account of random coil peaks ≈1650 cm^−1^. The region highlighted in light red is where the main change in composition occurs under the laser patterning. The bold curves represent the deduced absorbance band. The dotted curves represent the contributions to the amide I region (1580–1710 cm^−1^) and marked as random coil (R), β‐sheets (B), α‐helices (A), turns (T), and side chains (SC).

We suppose that the solubility change observed during low power laser exposure (<150 mW) originates from the melting of β‐sheet crystallites into SF's amorphous structure.^[^ ^36^ ^]^ The change in β‐sheet to α‐helix/random coil structures is a well‐known characteristic for the solubility of silk fibroin.^[^ ^1^, ^44^ ^]^ A quantitative study of the composition in silk fibroin is performed by Fourier transform infrared spectroscopy (FTIR). To obtain a sufficiently large sample area for the FTIR analysis, a 450 × 350 µm^2^ sized area was exposed with the DWL at a power of 150 mW and exposure time of 50 µs per indent. **Figure** **4** shows deconvoluted FTIR spectra of the amide I band before and after laser patterning of the SF thin film. We compare the results of the heat capacity measurements to the β‐pleated sheet content assessed by Fourier self‐deconvolution of the FTIR spectrum in the amide I region (1580–1710 cm^−1^). The spectrum of the annealing SF sample exhibits a peak ≈1620 cm^−1^ which is attributed to vibrational bands of β‐sheet structures. After the laser exposure, the intensity of β‐sheet bands decreases and the intensity of the random coil (R) band increases, which can be associated with the melting of β sheets and transition into amorphous phase.^[^ ^35^, ^36^ ^]^ From these measurements it can be concluded that a phase change has been induced due to the laser heating, which is in agreement with the experiment results in Figure 2a.

**Figure 5:**
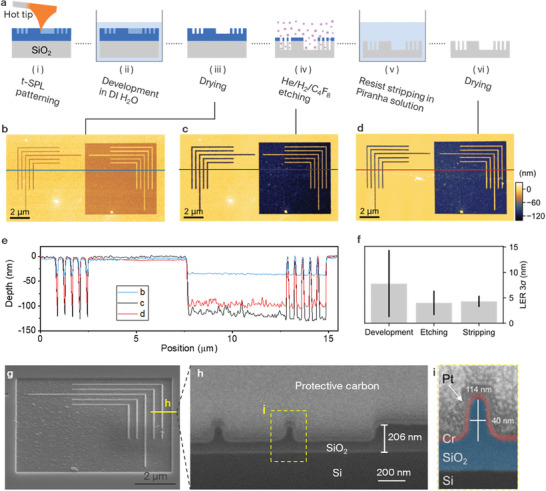
Dry etching of SiO_2_ using patterned SF resist as hard mask. a) Process flow of He/H_2_/C_4_F_8_ dry etching of SiO_2_ with a SF resist. b) Surface topography of a t‐SPL pattern in the resist after development. The initial thickness of the resist is 100 nm. c) Surface topography after He/H_2_/C_4_F_8_ dry etching for 40 s. d) Surface topography after stripping the SF away by immersion in a Piranha solution for 10 min. e) Surface profiles of patterned SF, etched SF and SiO_2_ as indicated by the line in panels b, c, and d. f) Line edge roughness (3σ) of line pattern after development, dry etching, and stripping. g) SEM image of the etched pattern in SiO_2_ observed at a tilt angle of 52°. h) SEM image of the cross‐section of the etched SiO_2_ prepared by FIB/SEM. i) Magnification of a line structure with false colors.

To transfer patterns written in SF resist into other materials such as SiO_2_, we demonstrate a fluorine chemistry‐based dry etching process of the resist as shown in **Figure** **5**a. The nanopatterns were prepared by the t‐SPL as discussed above. Then dry etching using mixed gases of He/H_2_/C_4_F_8_ was applied to a) remove the residual SF layer uniformly while retaining the pattern contrast, and b) to etch the SiO_2_ underneath where it was not protected by the SF film. Among the mixed gases, C_4_F_8_ gas is dissociated into plasma state by coupling RF power to it. By the bombarding ions, the long chains of the SF are broken into volatile parts. Subsequently, the bombarding ions also make the exposed SiO_2_ to break bonds. C_4_F_8_ in a plasma can generate many reactive species such as F atoms, radicals and ions, which are highly reactive and spontaneously attack SiO_2_ to produce volatile SiF_4_ and CO/CO_2_. Hydrogen gas is a radical scavenger to increase polymer formation and selectivity and reduce a bit etchant concentration.^[^ ^45^, ^46^ ^]^ Helium gas, as inert gas, is added to stabilize the plasma, dilute the chemical environment and enhance heat transfer from the sample.^[^ ^47^ ^]^ Additionally, helium addition causes inert ion bombardment of the surface, which assists in enhanced anisotropic etching. Finally, the patterned sample was treated with Piranha cleaning to strip remaining SF resist residues. Figure 5b shows the topography image of the SF resist pattern after development in DI water. Here two‐line arrays having a width of 50 nm are patterned in the SF resist with the depth of 30 nm. Figure 5c shows the result of dry etching using the patterned resist as a hard mask. The nanopattern written by the t‐SPL in the SF resist is transferred into the SiO_2_ layer by dry etching (Figure 5d). Figure 5e shows the trenches and the square that have been successfully transferred with an amplification of ≈3. The line edge roughness (3σ)^[^ ^48^ ^]^ decreases from the initial SF to the etched structure in SiO_2_ to a value of ≈5 ± 2 nm. The details of the SiO_2_ line nanostructure are imaged by SEM as shown in Figure 5g–i and Figure S8 (Supporting Information) of Supplementary Information. The line structures are 40 nm wide and 114 nm high.

To increase the selectivity between SF and SiO_2_, we performed studies where we varied the RF bias power. We found that decreasing the RF bias power reduces the etching rate of SF, while not significantly altering the one of SiO_2_. The details of the new different recipes are shown in **Table** **1** and Figure S7 (Supporting Information). We can vary the etching rate of SF using different dry etching chemistries to control the depth of the obtained patterns. At the RF bias power of 100 W, the etching rate of the SF is 4 nm min^−1^ and the etch selectivity between SiO_2_ and SF is as high as a remarkable value of 42:1. If the RF bias power decreases further, a higher selectivity can be obtained. However, it reaches a regime where the etching rate of SF drops drastically if the power is too low. It is also found that the etching rate of the SF is comparable to other commercial photoresists but is as low as ≈10% of the current standard resist for t‐SPL, polyphthalaldehyde (PPA)^[^ ^49^ ^]^ (details in Table S1, Supporting Information). Thus, we have shown a proof‐of‐principle, that silk fibroin in combination with thermal scanning probe lithography is a very capable resist for pattern writing and high‐aspect ratio transfer. Further optimization of the transfer parameters is still needed, such as residual layer etching. Sequential metal oxide vapor infiltration^[^ ^50^ ^]^ of silk fibroin can further increase the etch selectivity between the substrate and the resistor.

**Table 1:** Details of different recipes used for dry etching.

Recipe name	Chemistry flow rate [sccm]	Bias power [W]	SiO_2_ etching rate [nm min^−1^]	SF etching rate [nm min^−1^]	Selectivity SiO_2_: SF
SiO_2_ PR 5:1	He/H_2_/C_4_F_8_ (175/30/10)	300	215	134 ± 27	2:1
SiO_2_ PR 3:1	He/H_2_/C_4_F_8_ (175/18/15)	300	345	312.4 ± 1.7	1:1
SiO_2_ PR 3:1 soft	He/C_4_F_8_ (175/15)	150	295	258.7 ± 1.9	1:1
SiO_2_ PR 28:1 soft	He/H_2_/C_4_F_8_ (175/30/10)	100	166	4.0 ± 0.4	42:1

## 3 Conclusion

We demonstrate a novel method to perform lithography using silk fibroin as a positive tone resist, which exploits its polymorphic nature and solubility change under appropriate heating and solvent based annealing. The hybrid thermal lithography by DWL and t‐SPL shows the mix‐and‐match advantages of micropatterning by coarse writing and nanopatterning by fine writing with high alignment capability. Thanks to the very fast heating and cooling rates accessible by t‐SPL, nanopattern and grayscale pattern on the silk fibroin resist with a resolution down to 50 nm are possible. Further, the patterned resist is suitable as a hard mask for pattern transfer to other materials. 40 nm‐wide line structures in SiO_2_ are achieved using a dry etching step in fluorine chemistry yielding a high etch selectivity of 42:1 (SiO_2_: silk fibroin) with an excellent line edge roughness as low as 5 ± 2 nm. With the advantages of charged‐particle‐free t‐SPL method, this hybrid thermal lithography using bio‐friendly silk fibroin as resist and development in water is promising for high‐resolution green nanomanufacturing using more sustainable materials and processing systems.

## 4 Experimental Section

### Preparation of SF Resist

A 9.2 wt.% aqueous solution of SF was prepared from *Bombyx mori* silk cocoons using a standard procedure.^[^ ^40^ ^]^ The concentration was reduced to 1.8–3.1 wt.% for preparation of thinner films as detailed in Figure S6 (Supporting Information). SF was spin‐coated on silicon chips with a 200 nm‐thick SiO_2_ layer, which was treated with oxygen plasma at 100 W and 0.4 mbar for 1 min to improve surface wetting. The SF films were soft‐baked on a hot plate at 90 °C for 1 min to evaporate residual water. Subsequently, the samples were immersed in pure methanol (Fisher Chemical) for 4 min or in water vapor for 50 h to promote the formation of water‐insoluble β‐pleated sheet crystallites.^[^ ^29^ ^]^

### Hybrid Thermal Lithography Using SF

Micro/nanopatterning and grayscale patterning SF resistors were performed using a commercial t‐SPL integrated with direct write laser (NanoFrazor Explore, Heidelberg Instruments Nano, Switzerland). The cantilever was made of n‐doped silicon. The spring constant of the tip was ≈0.9 N m^−1^. The apex diameter is 15 ± 5 nm. The distance between indents (pixel size) was 20 nm. The indentation tip‐sample voltage is in the range of 4.5–9.5 V. Patterning was performed at a heater temperature of 650–960 °C.

### Pattern Transfer by Dry Etching

The SF patterns were directly transferred to 200 nm‐thick SiO_2_ layers simply by means of one‐step dry etching (SPTS APS Dielectric Etcher) with fluorine chemistry. For etching, the chips were affixed onto a 4‐inch silicon wafer by quick stick glue (temporary mounting wax). The substrate was kept at 10 °C to avoid overheating of the substrate. A composition of 175/30/10 sccm He/H_2_/C_4_F_8_ at a chamber pressure of 4 mTorr was used. Different parameters were investigated. The SF resist remaining on the protected regions was cleaned by O_2_ plasma for 1 min at 100 W and then immersed in Piranha solution for 10 min.

### Material characterizations

Topography images were taken either directly with the NanoFrazor or with a Bruker FastScan AFM (ScanAsyst‐Fluid + Probes). The SiO_2_ nanostructure sample was sputtered with a 12 nm thick chromium layer and 30 nm thick platinum layer for SEM observation. The lamella of the SiO_2_ nanostructure was cut by focused ion‐beam (FIB) milling (FEI Nova 600 NanoLab) and imaged by scanning electron microscopy (SEM) in the same tool. Fourier transform infrared (FTIR) spectra of annealed and thermally modified SF films were performed in attenuated total reflection (ATR) mode (Nicolet 6700, ThermoFisher Scientific). The thickness of the resist was measured by both mechanical profilometer (Bruker Dektak XT, surface profiler) and optical spectroscopic reflectometer (FilMetrics F54, Automated Thickness Mapping Systems).

## Conflict of Interest

The authors declare no conflict of interest.

## Supporting information

Supporting Information

## Data Availability

The data that support the findings of this study are available in the supplementary material of this article.
