# Supplementary material for: Multi‐ and Gray‐Scale Thermal Lithography of Silk Fibroin as Water‐Developable Resist for Micro and Nanofabrication
Source: Adv Sci (Weinh). 2024 Jan 17;11(12):2303518. doi: 10.1002/advs.202303518 (PMC10966559; doi:10.1002/advs.202303518)
Supplement: Supplementary file 1 — Supporting Information [file ADVS-11-2303518-s001.pdf]

ADVANCED SCIENCE
Open Access

Supporting Information

for *Adv. Sci.*, DOI 10.1002/advs.202303518

Multi- and Gray-Scale Thermal Lithography of Silk Fibroin as Water-Developable Resist for Micro and Nanofabrication

Mohammadreza Rostami, Aleksandra Marković, Ya Wang, Joffrey Pernollet, Xiaosheng Zhang, Xia Liu and Juergen Brugger*

Supplementary Information

Multi- and Gray-Scale Thermal Lithography of Silk Fibroin as Water-Developable Resist for Micro and Nanofabrication

Mohammadreza Rostami, Aleksandra Marković, Ya Wang, Joffrey Pernollet, Xiaosheng Zhang, Xia Liu[$,*], *Juergen Brugger*

M. Rostami, A. Marković, Y. Wang, X. Liu, J. Brugger
Microsystems Laboratory, Ecole Polytechnique Fédérale de Lausanne (EPFL), 1015 Lausanne, Switzerland
E-mail: xia.liu@bit.edu.cn (X. Liu)
[$]Current affiliation: School of Integrated Circuits and Electronics, MIIT Key Laboratory for Low-Dimensional Quantum Structure and Devices, Beijing Institute of Technology, Beijing 100081, China

J. Pernollet
Center for Micro and Nanotechnology (CMi), Ecole Polytechnique Fédérale de Lausanne (EPFL), 1015 Lausanne, Switzerland

X. Zhang
School of Electronic Science and Engineering, University of Electronic Science and Technology of China (UESTC), Chengdu 611731, China

Supplementary Section S1: SEM images of the cross-sections of the SF nanostructures

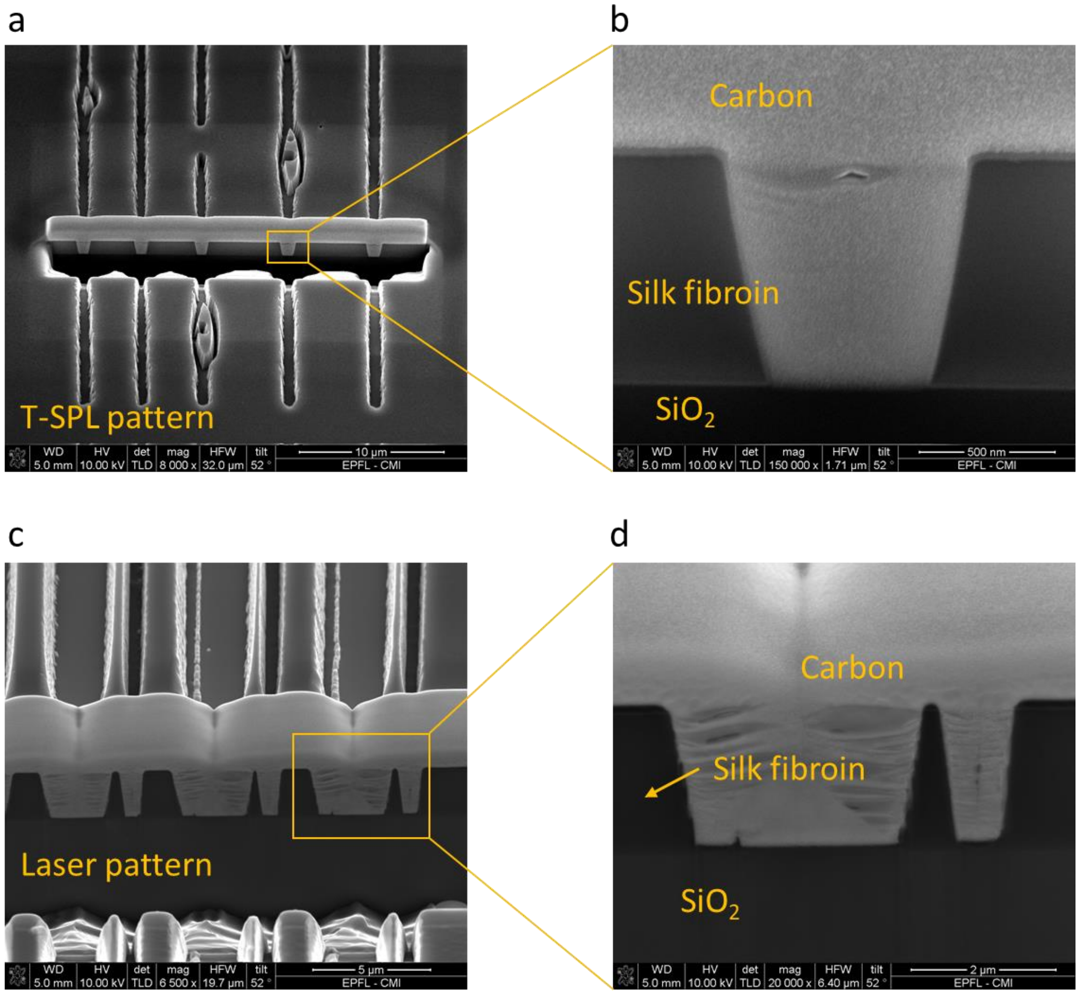

***Figure S1**: (a,b) Cross-sectional SEM images of the SF structure patterned by t-SPL. (c,d) Cross-sectional SEM images of the SF structure patterned by the laser. The samples were prepared by Ga^{+} ion beam milling using a dual beam FIB/SEM system.*

Supplementary Section S2: Grayscale pattern

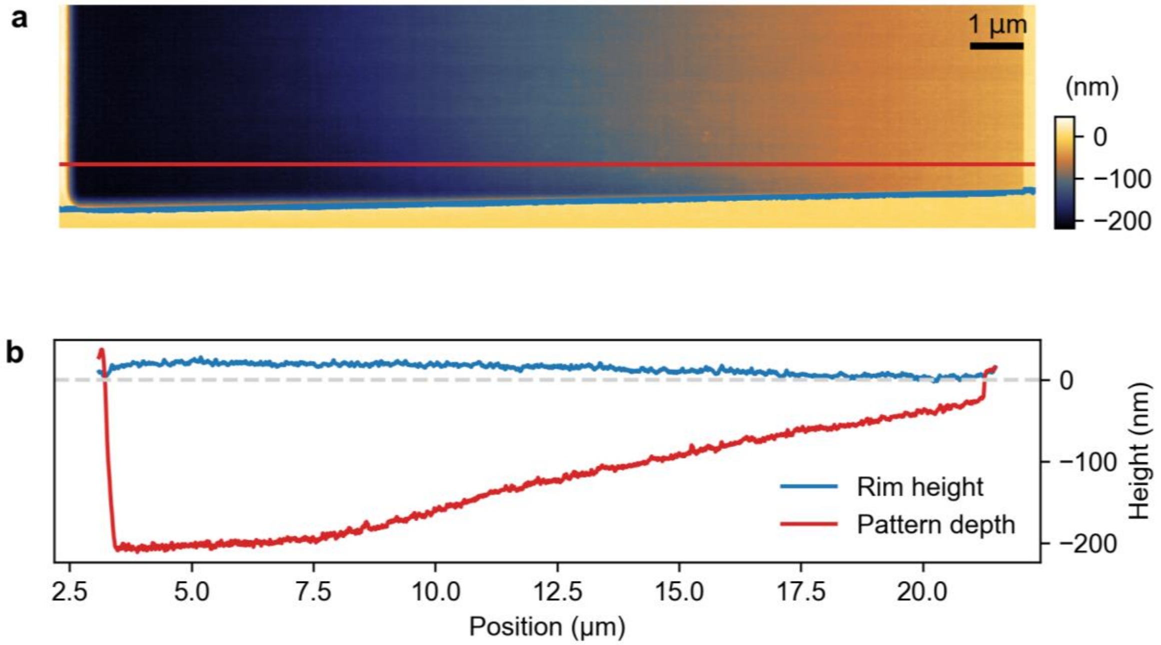

***Figure S2**: (a) The surface topography of a grayscale pattern with a linear gradient from 200 nm to 30 nm over 18 µm is shown. (b) The heights of the rim and the pattern are plotted as a function of the vertical position.*

Supplementary Section S3: Effect of power and exposure time on laser patterning

The silk fibroin resist was patterned by the DWL with longer pulse duration of 1 ms that is 50 times as shown in Figure S3 compared to that in Figure 1 and 2 of the main text. Significant topography change is made at the laser power of 50 mW, which suggests that the threshold laser power for patterning the resist decreases with longer exposure time. After the development in water, the line topography appears when the laser power is 5 mW (Figure S3b). The pattern depth increases with the laser power and saturates at laser power of 150 mW, as well as the pattern width, as shown in Figure S3c.

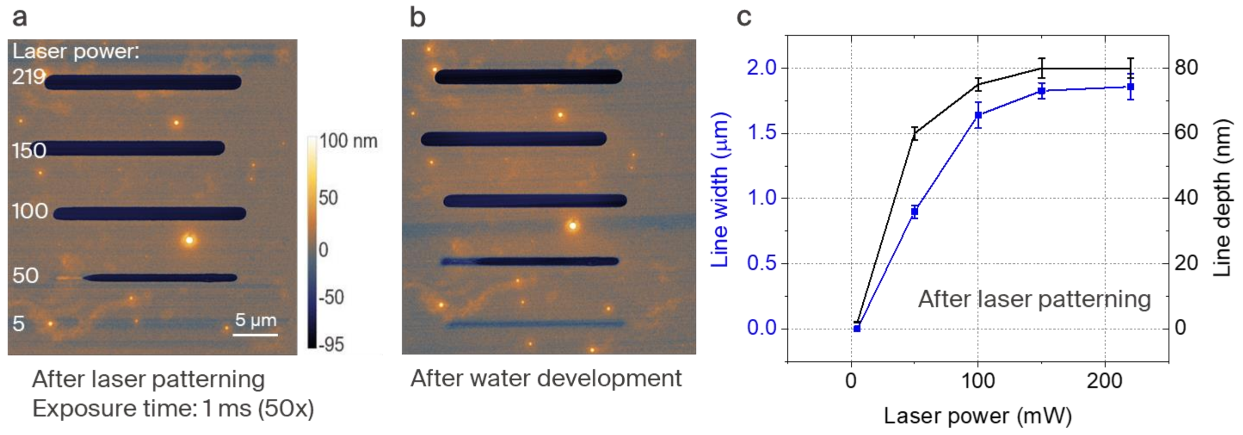

Figure S3: *(a) Straight lines fabricated by direct write laser (DWL) with different laser powers of 5, 50, 100, 150, and 219mW and increased exposure time of 1 ms that is 50 times compared to that in Figure 1 and 2 of the main text. (b) The image of line structures after the development in water. (c) Pattern widths and depths vary with the laser power at the increased exposure time of 1 ms.*

Supplementary Section S4: Tip temperature distribution and its effect on patterning silk fibroin resist

Figure S4a shows the simulation result of the temperature distribution around the thermal probe after 4 μs of heating. The contours in the graph indicate lines of equal temperature. The temperature is highest as 363 °C at the contact with the thermal probe, and drops as a function of distance. About 20-30 nm away from the tip, the temperature has dropped below T_g = 167-201 °C, the glass transition temperature of the silk fibroin. The melting temperature of β-crystallites (T_m = 292-351 °C), is only reached at a distance less than 3 nm away from the thermal probe. Figure S4b shows the heater temperature dependence of the pattern depth after t-SPL patterning and after development in water. Patterning was performed at a heater temperature of 650-800 °C. The silk fibroin resist was patterned by the t-SPL with short heat pulses of 20 μs resulting with slight surface modification with a depth less than 10 nm caused by the tip indentation. After the development in water, the thermally exposed resist dissolves, which shows in a clear topography contrast. The pattern depth increases with the heater temperature increasing.

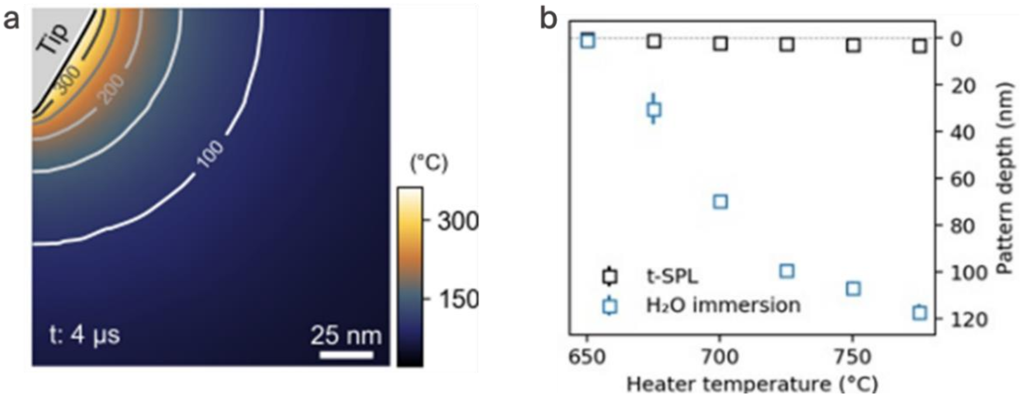

Figure S4*: (a) Simulated map of temperature distribution around the heated tip indenting the silk fibroin thin film after 4 μs of heating. (b) Pattern depths vary with the heater temperature after t-SPL patterning and after development in water.*

Supplementary Section S5: Comparison of the single t-SPL patterning process and the multiple t-SPL patterning process

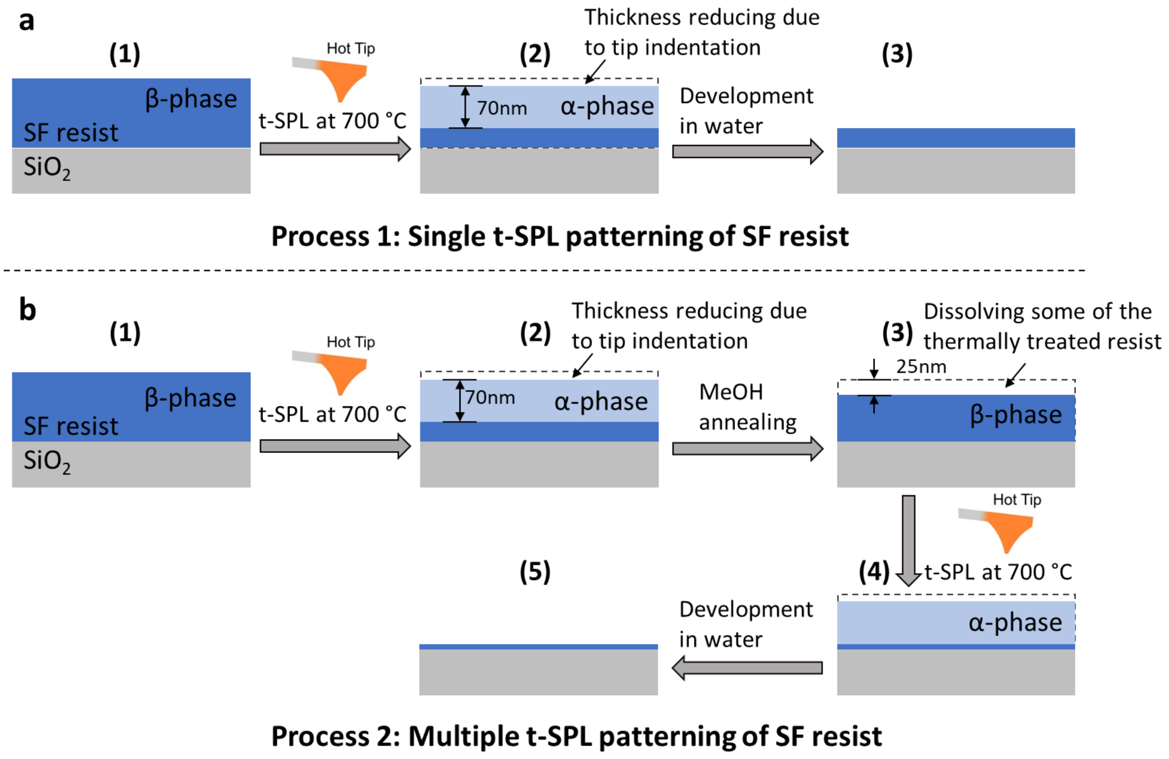

Figure S5: *Comparison of the single t-SPL patterning process and the multiple t-SPL patterning process.*

Supplementary Section S6: Spin coating and dry etching of silk fibroin

The concentration of the extracted silk fibroin from cocoons in water is about 9.2 wt%. Spin coating of silk fibroin with this concentration gives between 200-450 nm thick fibroin films. We obtained less than 100 nm thin film of silk fibroin required for the achievement of high-resolution patterning by dilution of silk fibroin solution. Figure S6 shows the variation of the silk fibroin resist thickness for different concentrations of silk fibroin solutions and spin speed. The thickness of the resist was measured both mechanically (Bruker Dektak XT, surface profiler) and optically (FilMetrics F54, Automated Thickness Mapping Systems) after spin coating and annealing in methanol.

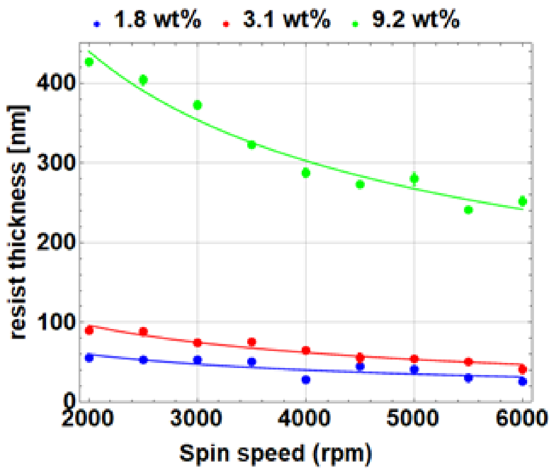

Figure S6*: The variation of the silk fibroin resist thickness with the concentration of silk fibroin solutions and spin speed.*

We determine the etching rate of silk fibroin in fluorine-based chemistry in Figure S7. The etching experiments were performed in a dry-chemistry etching chamber (SPTS APS, SPTS Technologies Ltd) using an inductively coupled plasma source for dry etching. The silk fibroin thin films were coated on 1cm × 1cm silicon chips. The chips were affixed onto a 4-inch silicon wafer by a quick stick to fit the holder in the tool. The substrate was kept at 10 °C to avoid overheating of the substrate. A standard gas mixture of He, H_2, and C_4F_8 was used for dry etching, which is known for a good etching selectivity of organic resists to SiO_2 at the pressure of 4 mTorr.

The etching rate of silk fibroin varies with different dry etching chemistries as shown in Figure S7b and S7c. The changes in the thickness of the resists after etching were measured by both mechanical profilometer (Bruker Dektak XT, surface profiler) and optical spectroscopic reflectometer (FilMetrics F54, Automated Thickness Mapping Systems). An etching rate was calculated from a least square fit to the measured data and then the etch selectivity between silk fibroin and SiO_2 was determined. Figure S7c shows the etching rate of the silk fibroin is lower than the current standard resist for t-SPL, poly-phthalaldehyde (PPA).

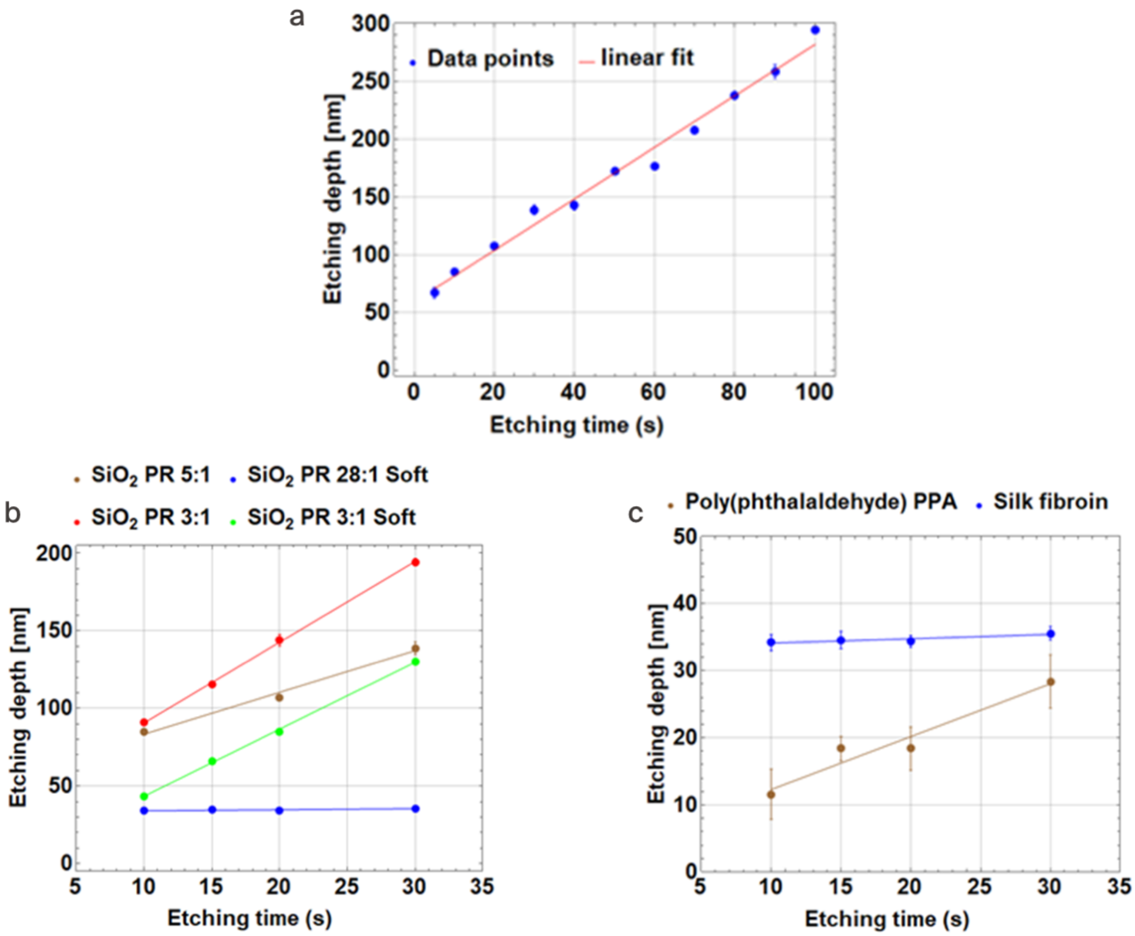

Figure S7: *(a) The etched depth of the silk fibroin film varies with etching time using the recipe called SiO_2 PR 5:1 (see Table 1). The line displays the linear regression of the data points to determine the etch rate of silk fibroin. (b) Comparison of the etching processes using different recipes. The line displays the linear regression of the data points to determine the etch rate of silk fibroin. (c) Comparison of the etching processes between the silk fibroin and PPA film using the softest etching chemistry called SiO_2 PR 28:1 soft. The line displays the linear regression of the data points to determine the etch rate of silk fibroin.*

We observed that silk fibroin and photo resist show a very large selectivity compared to SiO_2 for etching by a recipe called SiO_2 PR 28:1 soft (see Table 1). For this recipe, the bias power should be below the physical removal threshold of silk fibroin. For this softest etching recipe, we compared the etching rate of photoresist (PR, AZ9260 resist, MicroChemicals), PPA, and silk fibroin as shown in Table S1. The etching rate of silk fibroin is the lowest among the three resists. The selectivity values of SiO_2 compared to silk fibroin, PPA, and PR is about 42:1, 4:1, and 28:1, respectively.

***Table S1**: Etching rates of thermal and photo resists by the recipe called SiO_2 PR 28:1 soft in Table 1.*

**Resist name**	**Etching rate [nm/min]**
Polyphthalamide (PPA)	47.2 ± 2.9
Photoresist (PR)	6
Silk fibroin (SF)	4.0 ± 0.4

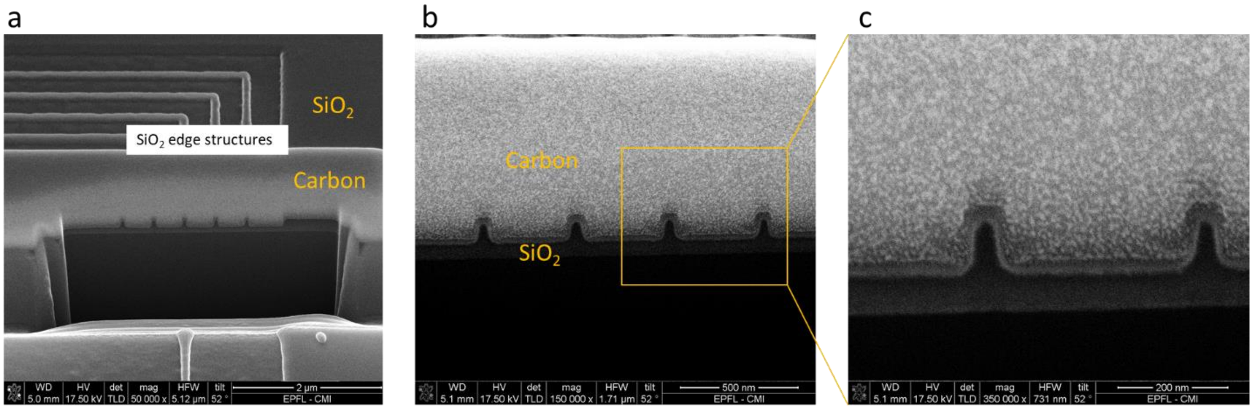

***Figure S8**: (a) Cross-sectional SEM image of the etched SiO_2 edge structures. (b,c) High-resolution SEM images of magnified SiO_2 structure.*
